# Therapeutic potential of berberine in cancer treatment: Current update

**DOI:** 10.17179/excli2026-9530

**Published:** 2026-07-03

**Authors:** Safwan Hazaa, Yachana Mishra, Vijay Mishra

**Affiliations:** 1 School of Pharmaceutical Sciences, Lovely Professional University, Phagwara (Punjab)-144411, India; 2 School of Bioengineering and Biosciences, Lovely Professional University, Phagwara (Punjab)-144411, India

## ⁯

Berberine (BBR) is a benzyl tetra isoquinoline alkaloid bioactive agent (Supplementary Figure 1), specifically 2,3-methylenedioxy-9,10-dimethoxy protoberberine chloride (C_20_H_18_NO_4_), with a calculated molecular weight of 336.36 g/mol. This compound is well-documented as a phytochemical constituent extracted and isolated from diverse botanical sources, such as Coptis rhizomes, *Pellodendron chenins*, and *Berberis vulgaris* (Rauf et al., 2021[2]). BBR exhibits noteworthy pharmacological activity against various diseases, inclusive of highly antidiabetic effects (Liang et al., 2019[1]), anti-inflammatory properties, anti-tumor activity, regulation of blood lipid and glucose levels, as well as potential roles in treatment of osteoporosis and osteoarthritis (Zhang et al., 2021[3]). Moreover, numerous studies have investigated the effects of berberine on various cancers, elucidating its underlying mechanisms of action. These investigations have led to a substantial body of literature encompassing diverse anticancer effects. Therefore, this letter aims to comprehensively address the therapeutic potential of berberine in the treatment of different types of cancer (Supplementary Table 1).

## Declaration

### Funding

None.

### Conflict of interest

The authors declare no conflict of interest.

### Artificial Intelligence (AI) - assisted technology

During the preparation of this letter, the authors used the Grammarly tool solely for language improvement, sentence structure enhancement, and grammar checking. The authors did not use AI tools for data analysis, interpretation, or the generation of scientific conclusions. After using this tool, the authors thoroughly reviewed, edited, and validated all content and take full responsibility for the accuracy, integrity, and scientific validity of the work. The authors assume full responsibility for the accuracy, originality, and scientific integrity of the final content of the published letter.

## Supplementary Material

Supplementary information
