# Supplementary material for: Therapeutic potential of berberine in cancer treatment: Current update
Source: EXCLI J. 2026 Jul 3;25:991–2. doi: 10.17179/excli2026-9530 (PMC13402736; doi:10.17179/excli2026-9530)
Supplement: Supplementary information [file EXCLI-25-991-s-001.pdf]

EXCLI Journal 2026;25:991-992 – ISSN 1611-2156
Received: May 01, 2026, accepted: June 04, 2026, published: July 03, 2026

Supplementary information to:

Letter to the editor:

THERAPEUTIC POTENTIAL OF BERBERINE IN CANCER TREATMENT: CURRENT UPDATE

Safwan Hazaa[1], Yachana Mishra[2*], Vijay Mishra[1]

[1] School of Pharmaceutical Sciences, Lovely Professional University, Phagwara (Punjab)-144411, India
[2] School of Bioengineering and Biosciences, Lovely Professional University, Phagwara (Punjab)-144411, India

* **Corresponding author:** Dr. Yachana Mishra, School of Bioengineering and Biosciences, Lovely Professional University, Phagwara (Punjab)-144411, India;
E-mail: yachanamishra@gmail.com

https://dx.doi.org/10.17179/excli2026-9530

This is an Open Access article distributed under the terms of the Creative Commons Attribution License (https://creativecommons.org/licenses/by/4.0/).

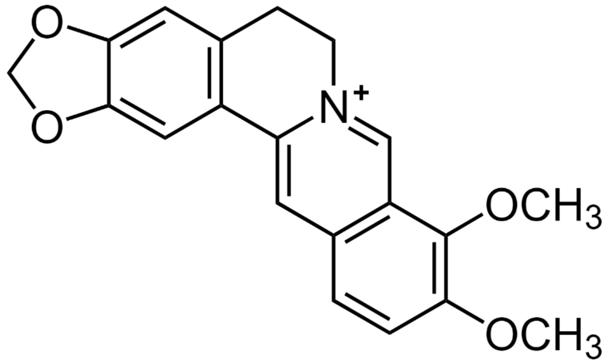

Supplementary Figure 1: Chemical structure of berberine

EXCLI Journal 2026;25:991-992 – ISSN 1611-2156
Received: May 01, 2026, accepted: June 04, 2026, published: July 03, 2026

Supplementary Table 1: Potential activity of berberine against various types of cancer

Type of cancer	Key findings	Reference
**Breast cancer**	Berberine's effect on MDA-MB-231 cells was studied, and the results revealed a dose-dependent reduction in cell viability and increased LDH release. BBR decreased colony formation and cell migration as well. Furthermore, it dramatically decreased the release of proinflammatory cytokines such as IL-1, IL-6, and TNF-. Furthermore, BBR therapy reduced expression of several proteins and mRNAs implicated in the NLRP3 inflammasome cascade, including P2X7, NLRP3, pro-caspase-1, ASC, caspase-1 p20, IL-18, and IL-1.	Yao et al., 2019
	The study explored the impact of BBR on the survival of Triple-Negative Breast Cancer (TNBC) cells at both cellular and molecular levels. The experimental model involved eight TNBC cell lines: MDA-MB-468, MDA-MB-231, HCC70, HCC38, HCC1937, HCC1143, BT-20, and BT-549. BBR exhibited cytotoxic effects on all treated TNBC cell lines, with HCC70 (IC_{50} = 0.19 µM), BT-20 (IC_{50} = 0.23 µM), and MDA-MB-468 (IC_{50} = 0.48 µM) being the most sensitive ones. BBR induced cell cycle arrest at G1 and/or G2/M phases and also triggered significant apoptosis. Remarkably, while BBR demonstrated cytotoxicity against TNBC cells, it had no effect on the viability of normal human breast cells (MCF10A) cultured in a 3D matrigel model.	El Khalki et al., 2020
	BBR exhibited a concentration-dependent suppression of cell proliferation in MDA-MB-468 (0, 3, 6, and 12 µM) and MDA-MB-231 (0, 6.25, 12.5, and 25 µM) cell lines. However, this inhibitory effect did not result from inducing cell apoptosis, necrosis, or autophagy. Cell cycle analysis revealed that BBR treatment led to an increased S+G2/M fraction in MDA-MB-231 and MDA-MB-453 cells, while in BBR-treated MDA-MB-468 and BT-549 cells, an increased G0/G1 fraction was observed. Further investigation demonstrated that BBR decreased the expression of Cyclin A and CDK1 in MDA-MB-231 and MDA-MB-453 cells.	Lin et al., 2019

Type of cancer	Key findings	Reference
**Liver cancer**	Asymmetric division of cell and polarized growth are regulated by the Par-3 Family Cell Polarity Regulator (PARD3) protein. PARD3 has a requisite role in mediating hepatic tumorigenesis. Overexpression of PARD3 accelerates liver tumor progression and positively correlates with high tumor stage as well as poor prognosis in cancer patients. PARD3 overexpressing activates the Sonic Hedgehog (SHH) signaling pathway through phosphorylation of SOX2, aPKC and Gli1, which associate with PARD3-overexpression. The report demonstrated that BBR is a potent tumorigenesis suppressor and preventive agent for hepatocellular cancer by inhibiting PARD3 expression.	Wu et al., 2024a
	The study proved that the chemotherapeutics-induced tumor cell apoptosis can also change the tumor microenvironment by activating the LOX pathway and subsequently release inflammatory factors such as LTB4, which can stimulate the adhesion and migration of the small number of surviving cells. BBR can reverse the adhesion and migration of HepG2 cells by inhibiting the expression of LOX-5 and reducing the LTB4 production in the tumor microenvironment.	Zhao et al., 2020
	BBR can suppress the expression of Cyclin D1 in human hepatic carcinoma cells both in vitro and in vivo. BBR inhibits Cyclin D1 expression in a dose- and time-dependent manner, and induces Cyclin D1 phosphorylation at Thr286 site, leading to its nuclear export and proteasomal degradation. BBR also recruits the SCFβ-TrCP complex to facilitate Cyclin D1 ubiquitin-proteasome dependent proteolysis. Knockdown of β-TrCP blocks Cyclin D1 turnover induced by BBR, and blocking the protein degradation induced by BBR in HepG2 cells increases tumor cell resistance to BBR. The authors suggested that BBR has potential as an anti-tumor agent for clinical cancer therapy.	Wang et al., 2016
	The study demonstrated notable anti-proliferative effects of BBR on Hep3B and BEL-7404 cells in in vitro tests. It achieved this by inhibiting SLC1A5, a transporter responsible for glutamine uptake. The increased expression of SLC1A5 led to enhanced glutamine uptake and improved resistance to BBR treatment. Berberine's inhibitory action on c-Myc was responsible for suppressing SLC1A5 expression. Additionally, in vivo experiments showed that BBR effectively suppressed tumor xenograft growth while also downregulating SLC1A5 and c-Myc expression. Notably, high SLC1A5 expression in hepatocellular carcinoma (HCC) tissues correlated with a poorer prognosis.	Zhang et al., 2019
	The study found that BBR deactivates the Akt pathway, which suppresses the S-phase kinase-associated protein 2 (Skp2) expression and enhances the expression and translocation of Forkhead box O3a (FoxO3a) into the nucleus. The translocated FoxO3a promotes the transcription of cyclin-dependent kinase inhibitors (CDKIs) p21Cip1 and p27Kip1, and represses Skp2 expression, leading to up-regulation of p21Cip1 and p27Kip1, causing G0/G1 phase cell cycle arrest in HCC.	Li et al., 2018

EXCLI Journal 2026;25:991-992 – ISSN 1611-2156
Received: May 01, 2026, accepted: June 04, 2026, published: July 03, 2026

Type of cancer	Key findings	Reference
**Colorectal cancer**	The study indicated that BBR treatment suppressed colorectal cancer cell viability by promoting apoptosis. LncRNA CASC2 was found to be upregulated in BBR-treated cells, and its knockdown reversed the BBR-induced apoptosis. Moreover, BBR treatment and lncRNA CASC2 suppressed the anti-apoptotic gene Bcl-2, leading to pro-apoptotic effects. The study further revealed that lncRNA CASC2 interacts with AUF1, sequestering it from binding to Bcl-2 mRNA, and thus inactivating Bcl-2 translation.	Dai et al., 2019
**Colon cancer**	BBR treatment demonstrated a suppressive effect on colon cancer cell viability and induced apoptosis along with increased caspase-3 activity in the human colon carcinoma HCT116 cell line. In this study, it was found that BBR downregulated miR-21 expression while promoting ITGβ4 and PDCD4 protein expression in HCT116 cells. Interestingly, overexpressing miR-21 counteracted the anti-cancer effects of BBR, leading to reduced cell viability, decreased apoptosis, and lower caspase-3 activity in the HCT116 cell line. However, overexpressing miR-21 also resulted in suppressed ITGβ4 and PDCD4 protein expression in the same cell line.	Lü et al., 2018
	This study demonstrated the regulatory effect of BBR on the intestinal microbiota and inhibition of colon cancer cell growth in vitro (HT29) and in vivo (nude mouse xenograft model) with HT29 colon cancer cells. The authors revealed that the inflammatory cytokine IL-10 levels ($P < 0.05$) were downregulated and DNMT1, c-Myc, and DNMT3B levels were reduced ($P < 0.05$) after berberine treatment, which significantly can lead to suppressed HT29 colon cancer cells proliferation. The report suggested that the inhibitory effect of BBR on colon cancer is related to metabolic function, DNMT regulation and intestinal microbiota.	Wang et al., 2023
**Lung cancer**	The study reported that BBR demonstrated potent suppression of NSCLC cell growth by triggering apoptosis in a dose- and time-dependent manner. It was evidenced by caspase-3 cleavage, cytochrome c release, and mitochondrial membrane depolarization in NSCLC cells. The induction of apoptosis by BBR was directly related to its concentration, and this effect involved sustained activation of c-jun-NH2-kinase (JNK). BBR-induced apoptosis was significantly reduced by the JNK inhibitor (SP600125).	Chen et al., 2022
	BBR does effectively suppress NSCLC cell proliferation and colony formation in vitro. In addition, it inhibited the growth of NSCLC tumors in subcutaneously transplanted tumor models, leading to extended survival in tumor-bearing mice. The anticancer effects of BBR were attributed to its ability to repress DNA repair and replication processes, as evidenced by the downregulation of genes involved in these pathways, such as RRM1, RRM2, LIG1, and POLE2, as revealed by cDNA microarray analysis.	Ni et al., 2022
**Esophageal cancer**	BBR inhibited the growth of esophageal cancer cells in a dose-dependent and time-dependent manner. KYSE-70 cells were more susceptible to the inhibitory activities of BBR than SKGT4 cells were. BBR treatment resulted in p21 up-regulation in KYSE-70 cells, inhibited the phosphorylation of Akt and p70S6K, and enhanced AMP-activated protein kinase phosphorylation in a sustained manner.	Jiang et al., 2017

EXCLI Journal 2026;25:991-992 – ISSN 1611-2156
Received: May 01, 2026, accepted: June 04, 2026, published: July 03, 2026

Type of cancer	Key findings	Reference
**Naso-pharyngeal cancer**	The study explored the potential cytotoxic effect of BBR on nasopharyngeal carcinoma (S18, C666-1, and 5-8F) cells, when the concentration of BBR is higher than 80 μM. BBR (≥ 80 μM) significantly increases lactate dehydrogenase (LDH) values, and shows a significant inhibitory effect in the proliferation, migration and invasive capacity in nasopharyngeal carcinoma cells. BBR induces autophagy by potentiating EGFR transcription. EGFR activates and regulates RAS–RAF1–MEK1/2–ERK1/2 signaling pathway via a specific super enhancer (SE) induction. Additionally, the knockdown of specific super enhancer markedly inhibits the expression of EGFR and phosphorylated EGFR (EGFR-p) in vitro, thereby reversing the inhibitory activity of BBR on nasopharyngeal carcinoma cells.	Wu et al., 2024b
	Ferroptosis process is a type of cell death and a critical pathway in tumor metastasis. BBR induces nasopharyngeal carcinoma cell death via ferroptosis. BBR is targeting and suppressing Xc-/GSH/GPX4 axis-driven system. For the first time, this study revealed that BBR induced ferroptosis in Nasopharyngeal carcinoma cells by increasing cellular Fe^{2+}, reactive oxygen species, and lipid peroxidation. Moreover, downregulation of GPX4 can directly or indirectly cause ferroptosis as a result of the inhibition of lipid peroxidation inhibitors. GPX4, lipid peroxidation key inhibitor is found to be significantly suppressed in BBR-treated Nasopharyngeal carcinoma cells.	Wu, et al., 2024c
**Ovarian cancer**	In this study, CCK8 assay, transwell assay, and wound healing assay were used to detect the proliferative capacity of SKOV3 cell invasion and estimate cell migration respectively. Quantitative real-time polymerase chain reaction (qRT-PCR) was employed to analyze the mRNA expression of miR-145 and MMP16. Western blot analysis was utilized to determine the protein level of MMP16. Furthermore, luciferase reporter assays were conducted to confirm miR-145's targeting of MMP16. The findings indicated that BBR effectively suppressed proliferation, migration, and invasion while enhancing miR-145 expression and reducing MMP16 expression in SKOV3 and 3AO cells. MiR-145 was confirmed to target MMP16. Furthermore, the observed reduction of MMP16 contributed to the inhibitory effects of BBR on proliferation, migration, and invasion.	Li et al., 2021

EXCLI Journal 2026;25:991-992 – ISSN 1611-2156
Received: May 01, 2026, accepted: June 04, 2026, published: July 03, 2026

Type of cancer	Key findings	Reference
**Brain cancer**	This study reveals the potent anti-inflammatory effects of BBR on glioma cells. BBR inhibits Caspase-1 activation through ERK1/2 signaling, resulting in reduced production of IL-1β and IL-18 by glioma cells. Furthermore, BBR treatment leads to decreased cell motility and subsequent cell death in U251 and U87 cells. Additionally, this research is the first to demonstrate that BBR can reverse epithelial-mesenchymal transition, a crucial marker of tumor invasion.	Tong et al., 2019
	The study revealed that BBR demonstrated significant inhibition of cell viability and proliferation in U251 and U87 glioblastoma cell lines, with IC_{50} values of 32 and 42 µmol/L, respectively. In addition, BBR at a concentration of 50 µmol/L reduced cell migration of HUVEC by 73 %. In an ectopic xenograft model, treatment with 50 mg/kg of BBR led to a substantial decrease in tumor weight (401.2±71.5 mg vs. 860.7±117.1 mg in the vehicle group). BBR also significantly reduced hemoglobin content (28.81±3.64 µg/mg vs. 40.84±5.15 µg/mg in the vehicle group), and CD31 mRNA expression in tumor tissue. Moreover, BBR effectively inhibited the phosphorylation of VEGFR2 and ERK ($p < 0.001$).	Jin et al., 2018
**Gastric cancer**	The paper investigates that BBR inhibits the proliferation, migration, and invasion of gastric cancer cells and suppresses tumor growth in vivo through the AMPK/HNF4α/WNT5A pathway. The knockdown of HNF4α in SGC7901 cells slowed cell proliferation, induced S phase arrest, and dramatically attenuated gastric cancer cells' metastasis and invasion. The anti-gastric cancer mechanism of BBR might be involved in the AMPK-HNF4α-WNT5A signaling pathway.	Hu et al., 2018
	BBR induced growth inhibition of gastric cancer cells without toxicity to human peripheral blood mononuclear cells. Treatment with BBR triggered cell autophagy, which confirmed the anti-cancer role of autophagy induced by BBR. Mechanistic studies showed that BBR inhibited mTOR, Akt and MAPK (ERK, JNK and p38) pathways, thereby inducing autophagy and cytotoxicity. Furthermore, inhibition of autophagy reversed BBR down-regulation, mTOR, Akt and MAPK. In xenografts, the BBR induced autophagy leads to suppression of tumor proliferation with no side-effect.	Zhang et al., 2020
**Prostate cancer**	The study explored berberine's potential as a therapeutic agent to delay the progression of castrate-resistant prostate cancer by targeting Aldo-keto reductase family 1 member C3 and reducing androgen synthesis. BBR effectively inhibited the proliferation of 22Rv1 prostate cancer cells and reduced cellular testosterone formation in a dose-dependent system. Notably, berberine's action was through inhibiting Aldo-keto reductase family 1 member C3 enzyme activity rather than affecting mRNA and protein expressions. Through a molecular docking study, it was observed that BBR could bind to the active center of Aldo-keto reductase family 1 member C3 and establish π-π interactions with specific amino acid residues, namely Phe306 and Phe311. This structural interaction accounted for the suppression of Aldo-keto reductase family 1 member C3 enzyme action and the inhibiting growth of cell 22Rv1 prostate cancer by reducing cellular androgen production.	Tian et al., 2016

Type of cancer	Key findings	Reference
**Pancreatic cancer**	BBR demonstrated a dose-dependent inhibition of cell growth by inducing cell cycle arrest and apoptosis. The treatment with BBR resulted in a 10 % increase in the G1 phase of PANC-1 cells and a 2 % increase in the G1 phase of MIA-PaCa2 cells, whereas gemcitabine primarily caused S-phase arrest for its antiproliferative effects. BBR-induced apoptosis in PANC-1 and MIA-PaCa2 cells was 7 % and 2 % higher than control cells, respectively. Notably, the apoptotic effect of BBR is more pronounced in PANC-1 cells compared to gemcitabine. Additionally, when PANC-1 and MIA-PaCa2 cells treated with BBR at its half-maximal inhibitory concentration (IC_{50}), apoptosis is induced through the generation of reactive oxygen species (ROS), rather than caspase 3/7 activation.	Park et al., 2015
	Through using bioinformatics analyses, mechanisms of BBR, and pharmacological activity investigation in silico, in vitro, and in vivo, BBR demonstrated that Berberine chloride suppresses proliferation of pancreatic adenocarcinoma (PAAD) via targeting four inflammatory related genes (PTGS2, CAPS3, ICAM1, and CXCR4). Four key targets docked well with BBR, revealed by using molecular docking-simulation. BBR exhibits a potential tumor-suppressive effect in xenografts.	Ruan et al., 2024
**Skin cancer**	This study explores the anti-metastasis effects of BBR on human melanoma cancer A375.S2 cells and A375.S2/PLX resistant cells in vitro. BBR was tested at low concentrations (0, 1, 1.5, and 2 μM) and was found to decrease in viable cell count, induce cell morphological changes, and inhibit mobility, migration, and invasion of A375.S2 cells. The study reported a slight inhibition of MMP-9 activity in A375.S2 cells due to BBR treatment. Furthermore, western blotting results indicated that BBR significantly reduced the expression of, MMP-13, E-cadherin, N-cadherin, p-ERK1/2, NF-κB, RhoA, ROCK1, SOS-1, MMP-1, GRB2, Ras, p-c-Jun, p-FAK, p-AKT, and uPA after 24 hours of treatment.	Liu et al., 2018
	The study found that BBR reduced the migration and invasion abilities of B16 cells and induced significant changes in epithelial-mesenchymal transition (EMT) factors related to pluripotency. BBR treatment decreased RARα, p-AKT, and p-PI3K expression while increasing RARβ and RARγ expression.	Kou et al., 2016

Received: May 01, 2026, accepted: June 04, 2026, published: July 03, 2026

Funding

None.

Conflict of interest

The authors declare no conflict of interest.

Artificial Intelligence (AI) – assisted technology

During the preparation of this letter, the authors used the Grammarly tool solely for language improvement, sentence structure enhancement, and grammar checking. The authors did not use AI tools for data analysis, interpretation, or the generation of scientific conclusions. After using this tool, the authors thoroughly reviewed, edited, and validated all content and take full responsibility for the accuracy, integrity, and scientific validity of the work. The authors assume full responsibility for the accuracy, originality, and scientific integrity of the final content of the published letter.

REFERENCES

Chen Q, Hou Y, Li D, Ding Z, Xu X, Hao B, et al. Berberine induces non-small cell lung cancer apoptosis via the activation of the ROS/ASK1/JNK pathway. Ann Transl Med. 2022;10:485.
doi: 10.21037/atm-22-1298

Dai W, Mu L, Cui Y, Li Y, Chen P, Xie H, et al. Berberine promotes apoptosis of colorectal cancer via regulation of the long non-coding RNA cancer susceptibility candidate 2/AU-binding factor 1/B-cell CLL/lymphoma 2 axis. Med Sci Monit. 2019;25:730–738.
doi: 10.12659/MSM.912082

El Khalki L, Maire V, Dubois T, Zyad A. Berberine impairs the survival of triple negative breast cancer cells: Cellular and molecular analyses. Molecules. 2020;25:506.
doi: 10.3390/molecules25030506

Hu Q, Li L, Zou X, Xu L, Yi P. Berberine attenuated proliferation, invasion and migration by targeting the AMPK/HNF4α/WNT5a pathway in gastric carcinoma. Front Pharmacol. 2018;9:1150.
doi: 10.3389/fphar.2018.01150

Jiang SX, Qi B, Yao WJ, Gu CW, Wei XF, Zhao Y, et al. Berberine displays antitumor activity in esophageal cancer cells in vitro. World J Gastroenterol. 2017;23: 2511–2518.
doi: 10.3748/wjg.v23.i14.2511

Jin F, Xie T, Huang X, Zhao X. Berberine inhibits angiogenesis in glioblastoma xenografts by targeting the VEGFR2/ERK pathway. Pharm Biol. 2018;56:665–671.
doi: 10.1080/13880209.2018.1548627

Kou Y, Li L, Li H, Tan Y, Li B, Wang K, et al. Berberine suppressed epithelial mesenchymal transition through cross-talk regulation of PI3K/AKT and RARα/RARβ in melanoma cells. Biochem Biophys Res Commun. 2016;479:290–296.
doi: 10.1016/j.bbrc.2016.09.061

Li F, Dong X, Lin P, Jiang J. Regulation of Akt/FoxO3a/Skp2 axis is critically involved in berberine-induced cell cycle arrest in hepatocellular carcinoma cells. Int J Mol Sci. 2018;19:327.
doi: 10.3390/ijms19020327

Li J, Zhang S, Wu L, Pei M, Jiang Y. Berberine inhibited metastasis through miR-145/MMP16 axis in vitro. J Ovarian Res. 2021;14:6.
doi: 10.1186/s13048-020-00752-2

Lin YS, Chiu YC, Tsai YH, Tsai YF, Wang JY, Tseng LM, et al. Different mechanisms involved in the berberine-induced antiproliferation effects in triple-negative breast cancer cell lines. J Cell Biochem. 2019;120: 13531–13544.
doi: 10.1002/jcb.28628

Liu JF, Lai KC, Peng SF, Maraming P, Huang YP, Huang AC, et al. Berberine inhibits human melanoma A375.S2 cell migration and invasion via affecting the FAK, uPA, and NF-κB signaling pathways and inhibits PLX4032 resistant A375.S2 cell migration in vitro. Molecules. 2018;23:2019.
doi: 10.3390/molecules23082019

Lü Y, Han B, Yu H, Cui Z, Li Z, Wang J. Berberine regulates the microRNA-21-ITGB4-PDCD4 axis and inhibits colon cancer viability. Oncol Lett. 2018;15: 5971–5976.
doi: 10.3892/ol.2018.7997

Ni L, Li Z, Ren H, Kong L, Chen X, Xiong M, et al. Berberine inhibits non-small cell lung cancer cell growth through repressing DNA repair and replication rather than through apoptosis. Clin Exp Pharmacol Physiol. 2022;49:134–144.
doi: 10.1111/1440-1681.13582

Park SH, Sung JH, Kim EJ, Chung N. Berberine induces apoptosis via ROS generation in PANC-1 and MIA-PaCa2 pancreatic cell lines. Braz J Med Biol Res. 2015;48:111–119.
doi: 10.1590/1414-431X20144293

Ruan LJ, Jiao JY, Cheng C, Zhang Y, Cao ZQ, He B, et al. Berberine chloride suppresses pancreatic adenocarcinoma proliferation and growth by targeting inflammation-related genes: An in silico analysis with in vitro and vivo validation. Cancer Chemother Pharmacol. 2024;94:169–181.
doi: 10.1007/s00280-024-04663-7

Tian Y, Zhao L, Wang Y, Zhang H, Xu D, Zhao X, et al. Berberine inhibits androgen synthesis by interaction with aldo-keto reductase 1C3 in 22Rv1 prostate cancer cells. Asian J Androl. 2016;18:607–612.
doi: 10.4103/1008-682X.169997

Tong L, Xie C, Wei Y, Qu Y, Liang H, Zhang Y, et al. Antitumor effects of berberine on gliomas via inactivation of caspase-1-mediated IL-1β and IL-18 release. Front Oncol. 2019;9:364.
doi: 10.3389/fonc.2019.00364

Wang N, Wang X, Tan HY, Li S, Tsang C, Tsao SW, et al. Berberine suppresses cyclin D1 expression through proteasomal degradation in human hepatoma cells. Int J Mol Sci. 2016;17:1899.
doi: 10.3390/ijms17111899

Wang X, Peng A, Huang C. Suppression of colon cancer growth by berberine mediated by the intestinal microbiota and the suppression of DNA methyltransferases (DNMTs). Mol Cell Biochem. 2023;479:2131–2141.
doi: 10.1007/s11010-023-04836-7

Wu J, Tan HY, Chan YT, Lu Y, Feng Z, Yuan H, et al. PARD3 drives tumorigenesis through activating Sonic Hedgehog signalling in tumour-initiating cells in liver cancer. J Exp Clin Cancer Res. 2024a;43:1–17.
doi: 10.1186/s13046-024-02967-3

Wu Y, Jia Q, Tang Q, Deng H, He Y, Tang F. Berberine-mediated ferroptosis through System Xc-/GSH/GPX4 axis inhibits metastasis of nasopharyngeal carcinoma. J Cancer. 2024b;15:685–698.
doi: 10.7150/jca.90574

Wu Y, Jia Q, Tang Q, Chen L, Deng H, He Y, et al. A specific super-enhancer actuated by berberine regulates EGFR-mediated RAS-RAF1-MEK1/2-ERK1/2 pathway to induce nasopharyngeal carcinoma autophagy. Cell Mol Biol Lett. 2024c;29:92.
doi: 10.1186/s11658-024-00607-4

Yao M, Fan X, Yuan B, Takagi N, Liu S, Han X, et al. Berberine inhibits NLRP3 inflammasome pathway in human triple-negative breast cancer MDA-MB-231 cell. BMC Complement Altern Med. 2019;19:216.
doi: 10.1186/s12906-019-2615-4

Zhang P, Wang Q, Lin Z, Yang P, Dou K, Zhang R. Berberine inhibits growth of liver cancer cells by suppressing glutamine uptake. Onco Targets Ther. 2019; 12:11751–11763.
doi: 10.2147/OTT.S235667

Zhang Q, Wang X, Cao S, Sun Y, He X, Jiang B, et al. Berberine represses human gastric cancer cell growth in vitro and in vivo by inducing cytostatic autophagy via inhibition of MAPK/mTOR/p70S6K and Akt signaling pathways. Biomed Pharmacother. 2020;128: 110245.
doi: 10.1016/j.biopha.2020.110245

Zhao Y, He K, Zheng H, Sun M, Shi T, Zheng X, et al. Berberine inhibits the apoptosis-induced metastasis by suppressing the iPLA2/LOX-5/LTB4 pathway in hepatocellular carcinoma. Onco Targets Ther. 2020;13: 5223–5230.
doi: 10.2147/OTT.S243357
